# Supplementary material for: Multi‐dataset identification of innovative feature genes and molecular mechanisms in keratoconus
Source: J Cell Mol Med. 2024 Sep 19;28(18):e70079. doi: 10.1111/jcmm.70079 (PMC11412914; doi:10.1111/jcmm.70079)
Supplement: Supplementary file 2 — Material S2. [file JCMM-28-e70079-s002.zip › 7.GSEA analysis/1.ARL11/KEGG_OLFACTORY_TRANSDUCTION.html]

Details for gene set KEGG\_OLFACTORY\_TRANSDUCTION[GSEA]

|  || Dataset | ARL11.group.cls#h\_versus\_l |
| Phenotype | group.cls#h\_versus\_l |
| Upregulated in class | l |
| GeneSet | KEGG\_OLFACTORY\_TRANSDUCTION |
| Enrichment Score (ES) | -0.34292218 |
| Normalized Enrichment Score (NES) | -1.1443678 |
| Nominal p-value | 0.12790698 |
| FDR q-value | 1.0 |
| FWER p-Value | 0.992 |
Table: GSEA Results Summary

  

Fig 1: Enrichment plot: KEGG\_OLFACTORY\_TRANSDUCTION      
 Profile of the Running ES Score & Positions of GeneSet Members on the Rank Ordered List

  

| SYMBOL | TITLE | RANK IN GENE LIST | RANK METRIC SCORE | RUNNING ES | CORE ENRICHMENT || 1 | OR10A2 | na | 51 | 0.750 | 0.0104 | No |
| 2 | OR2T6 | na | 244 | 0.559 | 0.0137 | No |
| 3 | OR4N4 | na | 547 | 0.466 | 0.0124 | No |
| 4 | OR2G2 | na | 623 | 0.451 | 0.0174 | No |
| 5 | OR6N2 | na | 743 | 0.425 | 0.0207 | No |
| 6 | OR4C6 | na | 1075 | 0.381 | 0.0171 | No |
| 7 | OR2C3 | na | 1078 | 0.380 | 0.0231 | No |
| 8 | OR6X1 | na | 1080 | 0.380 | 0.0291 | No |
| 9 | OR52H1 | na | 1202 | 0.366 | 0.0314 | No |
| 10 | OR2T1 | na | 1211 | 0.365 | 0.0369 | No |
| 11 | OR5T3 | na | 1259 | 0.359 | 0.0413 | No |
| 12 | OR9I1 | na | 1264 | 0.359 | 0.0469 | No |
| 13 | OR7A17 | na | 1287 | 0.358 | 0.0519 | No |
| 14 | OR13C3 | na | 1383 | 0.349 | 0.0547 | No |
| 15 | OR13C4 | na | 1406 | 0.346 | 0.0596 | No |
| 16 | OR10AD1 | na | 1415 | 0.345 | 0.0648 | No |
| 17 | OR6N1 | na | 1456 | 0.341 | 0.0691 | No |
| 18 | OR8B4 | na | 1486 | 0.338 | 0.0736 | No |
| 19 | OR6C74 | na | 1516 | 0.336 | 0.0781 | No |
| 20 | OR5P2 | na | 1727 | 0.318 | 0.0771 | No |
| 21 | OR8S1 | na | 1771 | 0.313 | 0.0808 | No |
| 22 | OR10T2 | na | 1916 | 0.303 | 0.0814 | No |
| 23 | OR1F1 | na | 1977 | 0.298 | 0.0844 | No |
| 24 | OR8K1 | na | 2092 | 0.289 | 0.0857 | No |
| 25 | OR6V1 | na | 2148 | 0.286 | 0.0886 | No |
| 26 | OR4S1 | na | 2341 | 0.274 | 0.0874 | No |
| 27 | OR5M8 | na | 2382 | 0.271 | 0.0906 | No |
| 28 | OR10K2 | na | 2416 | 0.270 | 0.0939 | No |
| 29 | OR10G9 | na | 2439 | 0.269 | 0.0975 | No |
| 30 | OR2G6 | na | 2479 | 0.266 | 0.1006 | No |
| 31 | OR7C1 | na | 2689 | 0.256 | 0.0986 | No |
| 32 | OR4S2 | na | 2754 | 0.253 | 0.1008 | No |
| 33 | OR52N1 | na | 2815 | 0.249 | 0.1030 | No |
| 34 | OR2M2 | na | 2844 | 0.248 | 0.1061 | No |
| 35 | OR13F1 | na | 2898 | 0.245 | 0.1085 | No |
| 36 | OR4X2 | na | 2954 | 0.243 | 0.1107 | No |
| 37 | OR52E6 | na | 2967 | 0.242 | 0.1142 | No |
| 38 | CALML6 | na | 3001 | 0.240 | 0.1171 | No |
| 39 | OR10V1 | na | 3185 | 0.233 | 0.1154 | No |
| 40 | OR6C65 | na | 3217 | 0.231 | 0.1182 | No |
| 41 | OR14C36 | na | 3359 | 0.224 | 0.1177 | No |
| 42 | OR5D13 | na | 3369 | 0.224 | 0.1210 | No |
| 43 | OR5B17 | na | 3404 | 0.223 | 0.1235 | No |
| 44 | OR10AG1 | na | 3448 | 0.221 | 0.1258 | No |
| 45 | CNGA3 | na | 3641 | 0.214 | 0.1236 | No |
| 46 | OR12D3 | na | 3663 | 0.213 | 0.1264 | No |
| 47 | OR2AG1 | na | 3721 | 0.211 | 0.1281 | No |
| 48 | OR13G1 | na | 3785 | 0.208 | 0.1296 | No |
| 49 | OR52K1 | na | 3879 | 0.205 | 0.1301 | No |
| 50 | OR9Q1 | na | 3900 | 0.204 | 0.1328 | No |
| 51 | OR5AS1 | na | 3907 | 0.204 | 0.1358 | No |
| 52 | OR4B1 | na | 3908 | 0.204 | 0.1391 | No |
| 53 | OR52A1 | na | 3925 | 0.203 | 0.1418 | No |
| 54 | CAMK2B | na | 3930 | 0.203 | 0.1450 | No |
| 55 | OR5T1 | na | 3974 | 0.201 | 0.1469 | No |
| 56 | OR10Q1 | na | 4192 | 0.194 | 0.1437 | No |
| 57 | OR4A16 | na | 4231 | 0.192 | 0.1456 | No |
| 58 | OR4K13 | na | 4470 | 0.184 | 0.1416 | No |
| 59 | OR5F1 | na | 4538 | 0.181 | 0.1426 | No |
| 60 | OR8K5 | na | 4576 | 0.180 | 0.1443 | No |
| 61 | OR5C1 | na | 4577 | 0.180 | 0.1472 | No |
| 62 | OR12D2 | na | 4722 | 0.175 | 0.1458 | No |
| 63 | OR2L3 | na | 4795 | 0.172 | 0.1464 | No |
| 64 | OR10A7 | na | 4811 | 0.172 | 0.1487 | No |
| 65 | CLCA1 | na | 4825 | 0.171 | 0.1511 | No |
| 66 | OR4P4 | na | 4899 | 0.169 | 0.1516 | No |
| 67 | OR9K2 | na | 5109 | 0.162 | 0.1481 | No |
| 68 | OR1D5 | na | 5149 | 0.161 | 0.1496 | No |
| 69 | OR1C1 | na | 5181 | 0.160 | 0.1512 | No |
| 70 | ADCY3 | na | 5276 | 0.158 | 0.1510 | No |
| 71 | CNGA4 | na | 5383 | 0.155 | 0.1504 | No |
| 72 | OR13C9 | na | 5443 | 0.153 | 0.1511 | No |
| 73 | OR2T8 | na | 5479 | 0.152 | 0.1525 | No |
| 74 | OR3A1 | na | 5602 | 0.148 | 0.1513 | No |
| 75 | OR8J1 | na | 5687 | 0.145 | 0.1512 | No |
| 76 | OR6C2 | na | 5817 | 0.142 | 0.1497 | No |
| 77 | OR4K15 | na | 6092 | 0.135 | 0.1438 | No |
| 78 | PDC | na | 6134 | 0.134 | 0.1448 | No |
| 79 | OR5I1 | na | 6135 | 0.133 | 0.1469 | No |
| 80 | OR10P1 | na | 6258 | 0.130 | 0.1454 | No |
| 81 | OR5K1 | na | 6303 | 0.129 | 0.1462 | No |
| 82 | OR6C75 | na | 6508 | 0.124 | 0.1422 | No |
| 83 | OR1L8 | na | 6574 | 0.122 | 0.1423 | No |
| 84 | OR52B2 | na | 6795 | 0.117 | 0.1377 | No |
| 85 | CAMK2A | na | 6879 | 0.114 | 0.1371 | No |
| 86 | OR7A5 | na | 6996 | 0.112 | 0.1355 | No |
| 87 | OR6T1 | na | 7007 | 0.112 | 0.1370 | No |
| 88 | OR6C76 | na | 7022 | 0.111 | 0.1384 | No |
| 89 | OR1M1 | na | 7048 | 0.111 | 0.1394 | No |
| 90 | OR1D2 | na | 7194 | 0.108 | 0.1369 | No |
| 91 | OR10H4 | na | 7198 | 0.108 | 0.1385 | No |
| 92 | OR52B6 | na | 7202 | 0.108 | 0.1401 | No |
| 93 | OR10G3 | na | 7239 | 0.107 | 0.1408 | No |
| 94 | OR56B4 | na | 7268 | 0.106 | 0.1417 | No |
| 95 | CLCA4 | na | 7302 | 0.105 | 0.1424 | No |
| 96 | OR51T1 | na | 7306 | 0.105 | 0.1440 | No |
| 97 | OR7G2 | na | 7427 | 0.103 | 0.1421 | No |
| 98 | OR4A47 | na | 7586 | 0.100 | 0.1391 | No |
| 99 | OR1J1 | na | 7714 | 0.098 | 0.1370 | No |
| 100 | OR2A2 | na | 7883 | 0.095 | 0.1336 | No |
| 101 | OR10X1 | na | 7911 | 0.094 | 0.1343 | No |
| 102 | OR1E1 | na | 7925 | 0.094 | 0.1354 | No |
| 103 | OR9A4 | na | 8070 | 0.091 | 0.1327 | No |
| 104 | OR4Q3 | na | 8182 | 0.089 | 0.1308 | No |
| 105 | OR1L4 | na | 8365 | 0.085 | 0.1269 | No |
| 106 | OR1A2 | na | 8882 | 0.077 | 0.1131 | No |
| 107 | OR5K2 | na | 8893 | 0.077 | 0.1140 | No |
| 108 | OR4C3 | na | 9179 | 0.072 | 0.1069 | No |
| 109 | OR2B11 | na | 9184 | 0.072 | 0.1079 | No |
| 110 | OR8G5 | na | 9409 | 0.069 | 0.1025 | No |
| 111 | OR3A2 | na | 9418 | 0.068 | 0.1033 | No |
| 112 | OR51E1 | na | 9560 | 0.066 | 0.1003 | No |
| 113 | OR2T5 | na | 9581 | 0.066 | 0.1008 | No |
| 114 | PRKX | na | 9610 | 0.066 | 0.1010 | No |
| 115 | OR14I1 | na | 9778 | 0.063 | 0.0971 | No |
| 116 | PRKG2 | na | 10052 | 0.060 | 0.0901 | No |
| 117 | OR52I1 | na | 10536 | 0.053 | 0.0769 | No |
| 118 | OR2F1 | na | 10877 | 0.049 | 0.0678 | No |
| 119 | OR2M7 | na | 10957 | 0.048 | 0.0663 | No |
| 120 | OR7D4 | na | 11192 | 0.045 | 0.0602 | No |
| 121 | CAMK2G | na | 11259 | 0.045 | 0.0590 | No |
| 122 | OR10C1 | na | 11314 | 0.044 | 0.0581 | No |
| 123 | OR10A4 | na | 11316 | 0.044 | 0.0588 | No |
| 124 | OR4D1 | na | 11352 | 0.043 | 0.0584 | No |
| 125 | OR4C11 | na | 11377 | 0.043 | 0.0584 | No |
| 126 | OR4D10 | na | 11394 | 0.043 | 0.0586 | No |
| 127 | OR56B1 | na | 11692 | 0.039 | 0.0506 | No |
| 128 | OR52I2 | na | 11694 | 0.039 | 0.0512 | No |
| 129 | OR2V2 | na | 11728 | 0.039 | 0.0509 | No |
| 130 | OR10G7 | na | 11915 | 0.037 | 0.0460 | No |
| 131 | OR1B1 | na | 11941 | 0.037 | 0.0459 | No |
| 132 | OR6K3 | na | 12052 | 0.036 | 0.0433 | No |
| 133 | OR5M9 | na | 12060 | 0.036 | 0.0436 | No |
| 134 | OR1N2 | na | 12110 | 0.035 | 0.0427 | No |
| 135 | OR4C46 | na | 12190 | 0.034 | 0.0410 | No |
| 136 | OR2T11 | na | 13231 | 0.025 | 0.0111 | No |
| 137 | OR10H2 | na | 13425 | 0.024 | 0.0059 | No |
| 138 | OR5AN1 | na | 13455 | 0.023 | 0.0054 | No |
| 139 | OR2L13 | na | 13756 | 0.021 | -0.0030 | No |
| 140 | OR7D2 | na | 13852 | 0.020 | -0.0054 | No |
| 141 | OR10H5 | na | 13859 | 0.020 | -0.0053 | No |
| 142 | OR11H4 | na | 14558 | 0.015 | -0.0254 | No |
| 143 | OR2A42 | na | 14574 | 0.014 | -0.0256 | No |
| 144 | CALM2 | na | 14606 | 0.014 | -0.0263 | No |
| 145 | OR52D1 | na | 14617 | 0.014 | -0.0263 | No |
| 146 | OR2D3 | na | 14684 | 0.014 | -0.0280 | No |
| 147 | OR52N4 | na | 14776 | 0.013 | -0.0305 | No |
| 148 | OR6A2 | na | 14802 | 0.013 | -0.0310 | No |
| 149 | OR2T10 | na | 14844 | 0.012 | -0.0320 | No |
| 150 | OR4X1 | na | 15021 | 0.011 | -0.0370 | No |
| 151 | OR2AK2 | na | 15308 | 0.009 | -0.0451 | No |
| 152 | OR4A5 | na | 15311 | 0.009 | -0.0451 | No |
| 153 | OR2B3 | na | 15320 | 0.009 | -0.0451 | No |
| 154 | OR6S1 | na | 15333 | 0.009 | -0.0454 | No |
| 155 | CLCA2 | na | 15550 | 0.007 | -0.0515 | No |
| 156 | OR5P3 | na | 16534 | -0.000 | -0.0801 | No |
| 157 | OR6K6 | na | 16601 | -0.001 | -0.0821 | No |
| 158 | OR52N2 | na | 16921 | -0.003 | -0.0913 | No |
| 159 | OR2A14 | na | 16951 | -0.003 | -0.0921 | No |
| 160 | OR52J3 | na | 17014 | -0.003 | -0.0938 | No |
| 161 | CALML5 | na | 17116 | -0.004 | -0.0967 | No |
| 162 | OR52E8 | na | 17276 | -0.005 | -0.1013 | No |
| 163 | OR51V1 | na | 17338 | -0.006 | -0.1030 | No |
| 164 | OR4C16 | na | 17503 | -0.007 | -0.1076 | No |
| 165 | OR52N5 | na | 17792 | -0.009 | -0.1159 | No |
| 166 | OR4E2 | na | 17896 | -0.010 | -0.1187 | No |
| 167 | OR52E4 | na | 17903 | -0.010 | -0.1187 | No |
| 168 | OR6F1 | na | 18228 | -0.012 | -0.1280 | No |
| 169 | OR6B2 | na | 18452 | -0.013 | -0.1343 | No |
| 170 | OR6B1 | na | 18475 | -0.013 | -0.1347 | No |
| 171 | CALML3 | na | 18646 | -0.015 | -0.1394 | No |
| 172 | OR10G8 | na | 18721 | -0.015 | -0.1413 | No |
| 173 | OR56A4 | na | 18736 | -0.015 | -0.1415 | No |
| 174 | OR6B3 | na | 18755 | -0.015 | -0.1418 | No |
| 175 | CALM1 | na | 18762 | -0.015 | -0.1417 | No |
| 176 | ARRB2 | na | 18772 | -0.015 | -0.1417 | No |
| 177 | OR51L1 | na | 18954 | -0.017 | -0.1467 | No |
| 178 | PRKACB | na | 18969 | -0.017 | -0.1468 | No |
| 179 | OR51F2 | na | 19213 | -0.018 | -0.1536 | No |
| 180 | OR4K1 | na | 19279 | -0.019 | -0.1552 | No |
| 181 | OR2G3 | na | 19300 | -0.019 | -0.1555 | No |
| 182 | OR4N5 | na | 19331 | -0.019 | -0.1561 | No |
| 183 | OR10A5 | na | 19433 | -0.020 | -0.1587 | No |
| 184 | OR10S1 | na | 19524 | -0.021 | -0.1610 | No |
| 185 | CNGB1 | na | 19862 | -0.023 | -0.1704 | No |
| 186 | OR4K5 | na | 20019 | -0.024 | -0.1746 | No |
| 187 | OR2T2 | na | 20032 | -0.024 | -0.1746 | No |
| 188 | OR5B21 | na | 20056 | -0.024 | -0.1748 | No |
| 189 | OR5J2 | na | 20310 | -0.026 | -0.1818 | No |
| 190 | OR8D4 | na | 20471 | -0.027 | -0.1860 | No |
| 191 | OR56A1 | na | 20648 | -0.029 | -0.1907 | No |
| 192 | OR5AK2 | na | 20679 | -0.029 | -0.1911 | No |
| 193 | OR4K2 | na | 20683 | -0.029 | -0.1907 | No |
| 194 | OR13C2 | na | 20890 | -0.030 | -0.1962 | No |
| 195 | OR5H2 | na | 20961 | -0.031 | -0.1978 | No |
| 196 | OR51A4 | na | 21127 | -0.032 | -0.2021 | No |
| 197 | OR6C6 | na | 21136 | -0.032 | -0.2018 | No |
| 198 | OR8I2 | na | 21319 | -0.034 | -0.2065 | No |
| 199 | OR2C1 | na | 21392 | -0.034 | -0.2081 | No |
| 200 | OR9A2 | na | 21649 | -0.036 | -0.2150 | No |
| 201 | OR1L6 | na | 21688 | -0.037 | -0.2155 | No |
| 202 | GUCY2D | na | 21711 | -0.037 | -0.2155 | No |
| 203 | GUCA1A | na | 21907 | -0.039 | -0.2206 | No |
| 204 | OR3A3 | na | 22177 | -0.042 | -0.2278 | No |
| 205 | OR8G1 | na | 22432 | -0.044 | -0.2345 | No |
| 206 | OR51Q1 | na | 22594 | -0.046 | -0.2384 | No |
| 207 | OR1L1 | na | 22706 | -0.047 | -0.2409 | No |
| 208 | OR5L2 | na | 22889 | -0.049 | -0.2454 | No |
| 209 | OR52K2 | na | 22920 | -0.049 | -0.2455 | No |
| 210 | CALM3 | na | 22971 | -0.050 | -0.2462 | No |
| 211 | GNAL | na | 23046 | -0.051 | -0.2475 | No |
| 212 | OR4C12 | na | 23194 | -0.052 | -0.2510 | No |
| 213 | PRKACA | na | 23665 | -0.058 | -0.2637 | No |
| 214 | OR2AG2 | na | 23692 | -0.058 | -0.2636 | No |
| 215 | OR10G4 | na | 23963 | -0.062 | -0.2704 | No |
| 216 | OR6M1 | na | 24121 | -0.064 | -0.2740 | No |
| 217 | OR5V1 | na | 24148 | -0.064 | -0.2737 | No |
| 218 | OR1J4 | na | 24296 | -0.066 | -0.2769 | No |
| 219 | OR51E2 | na | 24313 | -0.066 | -0.2763 | No |
| 220 | OR5M11 | na | 24685 | -0.072 | -0.2860 | No |
| 221 | OR51B4 | na | 24759 | -0.073 | -0.2870 | No |
| 222 | OR1S1 | na | 24769 | -0.073 | -0.2861 | No |
| 223 | OR52W1 | na | 24816 | -0.074 | -0.2862 | No |
| 224 | OR6K2 | na | 25120 | -0.078 | -0.2938 | No |
| 225 | OR4F6 | na | 25189 | -0.078 | -0.2946 | No |
| 226 | OR6Q1 | na | 25197 | -0.079 | -0.2935 | No |
| 227 | OR10A6 | na | 25223 | -0.079 | -0.2930 | No |
| 228 | OR2AE1 | na | 25340 | -0.081 | -0.2951 | No |
| 229 | OR5D18 | na | 25375 | -0.081 | -0.2948 | No |
| 230 | PRKACG | na | 25752 | -0.087 | -0.3043 | No |
| 231 | OR6C4 | na | 25930 | -0.089 | -0.3081 | No |
| 232 | OR5L1 | na | 26007 | -0.090 | -0.3089 | No |
| 233 | OR51A7 | na | 26039 | -0.091 | -0.3083 | No |
| 234 | OR11A1 | na | 26271 | -0.095 | -0.3135 | No |
| 235 | OR2M5 | na | 26289 | -0.095 | -0.3125 | No |
| 236 | OR51F1 | na | 26318 | -0.095 | -0.3118 | No |
| 237 | OR1A1 | na | 26498 | -0.099 | -0.3155 | No |
| 238 | OR2W1 | na | 26945 | -0.107 | -0.3267 | No |
| 239 | PDE1C | na | 27150 | -0.112 | -0.3309 | No |
| 240 | OR10Z1 | na | 27271 | -0.114 | -0.3326 | No |
| 241 | OR10H1 | na | 27460 | -0.118 | -0.3362 | No |
| 242 | OR13D1 | na | 27478 | -0.118 | -0.3348 | No |
| 243 | OR2J2 | na | 27483 | -0.118 | -0.3330 | No |
| 244 | OR10G2 | na | 27512 | -0.119 | -0.3319 | No |
| 245 | OR9Q2 | na | 27641 | -0.122 | -0.3337 | No |
| 246 | OR7E24 | na | 27694 | -0.123 | -0.3333 | No |
| 247 | OR4N2 | na | 27817 | -0.126 | -0.3348 | No |
| 248 | OR10J3 | na | 27935 | -0.129 | -0.3362 | No |
| 249 | OR4K14 | na | 28168 | -0.136 | -0.3408 | Yes |
| 250 | OR11G2 | na | 28178 | -0.136 | -0.3389 | Yes |
| 251 | OR51G1 | na | 28257 | -0.138 | -0.3389 | Yes |
| 252 | OR1K1 | na | 28259 | -0.138 | -0.3368 | Yes |
| 253 | OR6C68 | na | 28281 | -0.138 | -0.3352 | Yes |
| 254 | OR1I1 | na | 28415 | -0.141 | -0.3368 | Yes |
| 255 | OR4D6 | na | 28493 | -0.143 | -0.3368 | Yes |
| 256 | OR5B3 | na | 28509 | -0.144 | -0.3349 | Yes |
| 257 | OR51S1 | na | 28531 | -0.144 | -0.3332 | Yes |
| 258 | OR2T33 | na | 28614 | -0.147 | -0.3333 | Yes |
| 259 | OR2H1 | na | 28649 | -0.148 | -0.3319 | Yes |
| 260 | GUCA1B | na | 28719 | -0.150 | -0.3316 | Yes |
| 261 | OR2T34 | na | 28786 | -0.152 | -0.3311 | Yes |
| 262 | OR14A16 | na | 28794 | -0.152 | -0.3289 | Yes |
| 263 | OR10K1 | na | 28899 | -0.154 | -0.3294 | Yes |
| 264 | OR10A3 | na | 28984 | -0.156 | -0.3294 | Yes |
| 265 | OR4D11 | na | 29003 | -0.156 | -0.3274 | Yes |
| 266 | OR52L1 | na | 29087 | -0.159 | -0.3273 | Yes |
| 267 | OR7C2 | na | 29088 | -0.159 | -0.3248 | Yes |
| 268 | OR2Z1 | na | 29132 | -0.160 | -0.3235 | Yes |
| 269 | OR2L8 | na | 29134 | -0.160 | -0.3210 | Yes |
| 270 | OR2B2 | na | 29310 | -0.166 | -0.3234 | Yes |
| 271 | OR13J1 | na | 29360 | -0.168 | -0.3222 | Yes |
| 272 | OR1J2 | na | 29446 | -0.171 | -0.3220 | Yes |
| 273 | OR51B5 | na | 29570 | -0.174 | -0.3228 | Yes |
| 274 | OR13C8 | na | 29591 | -0.175 | -0.3206 | Yes |
| 275 | OR5A1 | na | 29613 | -0.176 | -0.3184 | Yes |
| 276 | OR2W3 | na | 29655 | -0.177 | -0.3167 | Yes |
| 277 | OR4C13 | na | 29709 | -0.179 | -0.3154 | Yes |
| 278 | OR5M10 | na | 29867 | -0.183 | -0.3171 | Yes |
| 279 | OR8A1 | na | 29948 | -0.186 | -0.3165 | Yes |
| 280 | OR2B6 | na | 29974 | -0.187 | -0.3142 | Yes |
| 281 | GUCA1C | na | 30078 | -0.190 | -0.3142 | Yes |
| 282 | OR5D14 | na | 30136 | -0.192 | -0.3128 | Yes |
| 283 | OR8D1 | na | 30255 | -0.196 | -0.3131 | Yes |
| 284 | OR9G1 | na | 30259 | -0.196 | -0.3101 | Yes |
| 285 | OR5M1 | na | 30403 | -0.201 | -0.3111 | Yes |
| 286 | OR7G3 | na | 30461 | -0.203 | -0.3095 | Yes |
| 287 | OR8H1 | na | 30475 | -0.203 | -0.3067 | Yes |
| 288 | OR51A2 | na | 30516 | -0.205 | -0.3046 | Yes |
| 289 | OR2M3 | na | 30520 | -0.205 | -0.3014 | Yes |
| 290 | OR10R2 | na | 30535 | -0.205 | -0.2985 | Yes |
| 291 | OR2M4 | na | 30537 | -0.205 | -0.2953 | Yes |
| 292 | OR2J3 | na | 30589 | -0.207 | -0.2935 | Yes |
| 293 | OR8B8 | na | 30602 | -0.208 | -0.2905 | Yes |
| 294 | OR2A12 | na | 30617 | -0.208 | -0.2876 | Yes |
| 295 | OR6Y1 | na | 30707 | -0.211 | -0.2869 | Yes |
| 296 | OR1N1 | na | 30739 | -0.212 | -0.2844 | Yes |
| 297 | OR51G2 | na | 30855 | -0.216 | -0.2843 | Yes |
| 298 | OR4D9 | na | 30915 | -0.218 | -0.2826 | Yes |
| 299 | OR4D5 | na | 30979 | -0.220 | -0.2809 | Yes |
| 300 | OR52A5 | na | 31071 | -0.223 | -0.2800 | Yes |
| 301 | OR5AU1 | na | 31106 | -0.224 | -0.2774 | Yes |
| 302 | OR5AR1 | na | 31120 | -0.225 | -0.2742 | Yes |
| 303 | OR7G1 | na | 31124 | -0.225 | -0.2707 | Yes |
| 304 | OR51B2 | na | 31194 | -0.228 | -0.2691 | Yes |
| 305 | OR11L1 | na | 31204 | -0.228 | -0.2658 | Yes |
| 306 | OR5B12 | na | 31211 | -0.228 | -0.2623 | Yes |
| 307 | OR6C3 | na | 31221 | -0.229 | -0.2589 | Yes |
| 308 | OR10J5 | na | 31346 | -0.234 | -0.2588 | Yes |
| 309 | OR4F15 | na | 31380 | -0.235 | -0.2561 | Yes |
| 310 | OR5D16 | na | 31401 | -0.236 | -0.2529 | Yes |
| 311 | OR2H2 | na | 31409 | -0.236 | -0.2493 | Yes |
| 312 | OR52M1 | na | 31417 | -0.236 | -0.2458 | Yes |
| 313 | OR4A15 | na | 31521 | -0.240 | -0.2450 | Yes |
| 314 | OR4D2 | na | 31590 | -0.243 | -0.2431 | Yes |
| 315 | OR6C70 | na | 31730 | -0.249 | -0.2432 | Yes |
| 316 | OR6C1 | na | 31794 | -0.252 | -0.2410 | Yes |
| 317 | OR1G1 | na | 31810 | -0.253 | -0.2374 | Yes |
| 318 | OR52B4 | na | 31967 | -0.259 | -0.2378 | Yes |
| 319 | OR56A5 | na | 31979 | -0.259 | -0.2340 | Yes |
| 320 | OR5M3 | na | 32126 | -0.266 | -0.2340 | Yes |
| 321 | OR5AP2 | na | 32132 | -0.266 | -0.2300 | Yes |
| 322 | CAMK2D | na | 32170 | -0.268 | -0.2268 | Yes |
| 323 | OR5AC2 | na | 32205 | -0.269 | -0.2235 | Yes |
| 324 | OR2AT4 | na | 32252 | -0.272 | -0.2205 | Yes |
| 325 | OR4C45 | na | 32331 | -0.276 | -0.2184 | Yes |
| 326 | OR4L1 | na | 32437 | -0.283 | -0.2169 | Yes |
| 327 | OR1S2 | na | 32456 | -0.284 | -0.2129 | Yes |
| 328 | OR2F2 | na | 32540 | -0.290 | -0.2107 | Yes |
| 329 | OR8D2 | na | 32604 | -0.294 | -0.2079 | Yes |
| 330 | OR4C15 | na | 32618 | -0.295 | -0.2036 | Yes |
| 331 | OR1L3 | na | 32623 | -0.296 | -0.1990 | Yes |
| 332 | OR2T4 | na | 32661 | -0.299 | -0.1953 | Yes |
| 333 | OR11H6 | na | 32813 | -0.308 | -0.1948 | Yes |
| 334 | OR14J1 | na | 32873 | -0.313 | -0.1916 | Yes |
| 335 | OR51I1 | na | 32888 | -0.313 | -0.1870 | Yes |
| 336 | OR13H1 | na | 32906 | -0.315 | -0.1825 | Yes |
| 337 | OR51M1 | na | 32979 | -0.320 | -0.1795 | Yes |
| 338 | OR2A5 | na | 32987 | -0.321 | -0.1746 | Yes |
| 339 | OR13C5 | na | 33014 | -0.323 | -0.1702 | Yes |
| 340 | OR2S2 | na | 33064 | -0.326 | -0.1664 | Yes |
| 341 | OR4K17 | na | 33285 | -0.343 | -0.1674 | Yes |
| 342 | OR2T12 | na | 33300 | -0.344 | -0.1623 | Yes |
| 343 | OR56A3 | na | 33314 | -0.345 | -0.1572 | Yes |
| 344 | OR8K3 | na | 33319 | -0.345 | -0.1518 | Yes |
| 345 | OR8U8 | na | 33351 | -0.348 | -0.1472 | Yes |
| 346 | OR13A1 | na | 33365 | -0.349 | -0.1420 | Yes |
| 347 | OR2T27 | na | 33428 | -0.354 | -0.1382 | Yes |
| 348 | OR2K2 | na | 33518 | -0.361 | -0.1350 | Yes |
| 349 | OR1Q1 | na | 33537 | -0.363 | -0.1298 | Yes |
| 350 | OR2D2 | na | 33707 | -0.380 | -0.1287 | Yes |
| 351 | OR8U1 | na | 33748 | -0.384 | -0.1237 | Yes |
| 352 | OR5H6 | na | 33757 | -0.384 | -0.1179 | Yes |
| 353 | OR4M1 | na | 33779 | -0.387 | -0.1123 | Yes |
| 354 | OR2A7 | na | 33797 | -0.389 | -0.1066 | Yes |
| 355 | OR52R1 | na | 33862 | -0.399 | -0.1021 | Yes |
| 356 | PRKG1 | na | 33894 | -0.404 | -0.0966 | Yes |
| 357 | OR8H3 | na | 33934 | -0.410 | -0.0912 | Yes |
| 358 | OR52E2 | na | 33973 | -0.416 | -0.0857 | Yes |
| 359 | OR7A10 | na | 34069 | -0.429 | -0.0817 | Yes |
| 360 | OR8B12 | na | 34072 | -0.430 | -0.0749 | Yes |
| 361 | OR10J1 | na | 34077 | -0.431 | -0.0682 | Yes |
| 362 | OR51B6 | na | 34085 | -0.433 | -0.0615 | Yes |
| 363 | OR2A25 | na | 34165 | -0.447 | -0.0567 | Yes |
| 364 | OR8H2 | na | 34269 | -0.467 | -0.0522 | Yes |
| 365 | OR2L2 | na | 34281 | -0.470 | -0.0451 | Yes |
| 366 | OR5T2 | na | 34322 | -0.479 | -0.0386 | Yes |
| 367 | OR51I2 | na | 34331 | -0.482 | -0.0312 | Yes |
| 368 | OR51D1 | na | 34333 | -0.483 | -0.0235 | Yes |
| 369 | OR9G4 | na | 34344 | -0.485 | -0.0161 | Yes |
| 370 | OR5A2 | na | 34352 | -0.487 | -0.0086 | Yes |
| 371 | OR2Y1 | na | 34490 | -0.531 | -0.0041 | Yes |
| 372 | OR8J3 | na | 34674 | -0.693 | 0.0016 | Yes |
Table: GSEA details [plain text format]

  

Fig 2: KEGG\_OLFACTORY\_TRANSDUCTION      
 Blue-Pink O' Gram in the Space of the Analyzed GeneSet

  

Fig 3: KEGG\_OLFACTORY\_TRANSDUCTION: Random ES distribution      
 Gene set null distribution of ES for **KEGG\_OLFACTORY\_TRANSDUCTION**
